# Is Physical Intimate Partner Violence a Risk Factor for Physical Child Maltreatment in a Nationally Representative Sample of Finnish School Children?

**DOI:** 10.1177/08862605241289476

**Published:** 2024-10-23

**Authors:** Laura Annika Mielityinen, Noora Ellonen, Monica Fagerlund

**Affiliations:** 1 Tampere University, Finland

**Keywords:** physical child maltreatment, physical intimate partner violence, co-occurrence, risk factors, prevention

## Abstract

Previous studies have found a high co-occurrence between intimate partner violence and child maltreatment. However, little is known about the nuanced association between physical intimate partner violence (pIPV) and physical child maltreatment (pCM) in countries where corporal punishment is prohibited by law. Furthermore, there is a lack of information on the co-occurrence from children’s perspectives and nationally representative surveys. The main objective of this study was to examine the connection between pIPV and pCM in a nationally representative sample of 12 to 13 and 15- to 16-year-old Finnish children (*N* = 6,825) after controlling for other known risk factors. The χ^2^ test and the logistic regression model were used. Around 47.3% of the children who had been exposed to pIPV had also experienced pCM during the past year, whereas 6.7% of those who had not been exposed to pIPV reported pCM. Children exposed to pIPV were almost three times more likely to experience pCM than children who were not exposed to pIPV. The connection remained statistically significant after controlling for other risk factors. Prevention and early identification of pIPV might reduce pCM in families. Targeted prevention efforts and interventions aimed at physical family violence are necessary to reduce its occurrence and mitigate the impact of abuse on children and families.

Intimate partner violence (IPV) and child maltreatment are global public health concerns (Chan et al., 2021; Gilbert et al., 2009; Lee & Kim, 2023; Mielityinen et al., 2023). Over the last decade, there has been an increase in research examining the connection between these two forms of family violence, as they often co-occur (Ahmadabadi et al., 2018; Casanueva et al., 2009; MacDonell, 2012; Sijtsema et al., 2020; Skafida et al., 2022). In families where these two forms of family violence coexist, children see or hear the violence between guardians or are exposed to seeing the aftermath (bruises) in addition to being maltreated themselves (Jouriles et al., 2008). Co-occurring IPV and child maltreatment can be considered forms of poly-victimization because they represent multiple, overlapping types of victimization that affect a child simultaneously or within a specific timeframe (Finkelhor et al., 2007; Miranda et al., 2021).

Although previous research has emphasized the need to examine multiple overlapping forms of violence *(*Ahmadabadi et al., 2018; Finkelhor et al., 2007; Sijtsema et al., 2020), several international studies highlight the importance of differentiating between emotional and physical family violence in research, as well as in prevention and intervention efforts (Afifi et al., 2012; Gilbert et al., 2009; Miranda et al., 2021). Although physical violence rarely happens in families without emotional violence, there is a significant amount of emotional violence without physical violence (Sijtsema et al., 2020). This underscores the need for nuanced analyses and interpretations of research findings, as understanding the risk factors, dynamics, and consequences specific to each type of maltreatment enables targeted prevention efforts and interventions aimed at reducing the occurrence and mitigating the impact of abuse and neglect on children and families (October & Laitinen, 2022; Rodriguez et al., 2016).

Furthermore, insight into the co-occurrence of physical IPV (pIPV) and physical child maltreatment (pCM) is highly relevant since their exposure during early life poses a serious public health issue (Guedes et al., 2016; Sijtsema et al., 2020). Beyond immediate physical harm, pCM has several long-term effects and consequences, such as the risks of poorer physical health, substance abuse, juvenile delinquency, suicidality, and psychiatric disorders (Gilbert et al., 2009; Graf et al., 2021; Hughes et al., 2017; Yohros, 2023). The psychological, physical, social, and cognitive consequences of exposure to pIPV on a child are similar to the consequences of pCM and are substantial, even if no child maltreatment is involved (Harper et al., 2018; Howell et al., 2016; MacDonell, 2012).

Moreover, research evidence shows that co-occurring pIPV and pCM are, similar to poly-victimization, more strongly associated with trauma symptoms, behavioral problems, and poorer health than experiencing these types of violence separately (Finkelhor et al., 2007; Guedes et al., 2016; Hamby et al., 2010). In addition, children raised in homes with both pIPV and pCM are at higher risk of perpetrating or becoming victims of family violence later in life (Cochran et al., 2017; Copp et al., 2019; Hou et al., 2016). While previous research has focused on the transmission of single forms of family violence, such as child maltreatment, recent studies emphasize the importance of examining the nuanced relationships between different types of family violence across two generations to better understand intergenerational pathways (Buchanan et al., 2023; Haselschwerdt et al., 2019).

Despite its relevance, studying the connection between pIPV and pCM and drawing conclusions from previous results is complex for several reasons. First, there is vast variation in the operationalization of IPV (Buchanan et al., 2023). Many studies examining co-occurrence combine the acts of physical and emotional IPV (Ahmadabadi et al., 2018; Castro et al., 2017; Skafida et al., 2022). Therefore, it is difficult to specify whether it is physical or emotional IPV that is connected to pCM or whether physical and emotional violence has an equal connection with pCM. Second, there is also variation in the definition of pCM (Laajasalo et al., 2023).

In addition, the data present challenges to examining pIPV and pCM and specifically to examining of their co-occurrence. Many studies have used limited samples from child protection services and other social service authorities. This typically leads to higher prevalence rates and higher odds ratios than in the general population since these families often have multiple problems and few resources compared to the general population (Chan et al., 2021; Mathews et al., 2022; Tiyyagura et al., 2018). The sample of these studies is thus biased, and the findings cannot be generalized to a larger population.

Administrative register data have been suggested as a valuable source for exploring different types of violence in the home and avoiding selection biases related to clinical samples and survey data (Ellonen et al., 2024; Fluke et al., 2021; Soneson et al., 2023). However, both pIPV and pCM tend to occur behind closed doors and do not stand out in official records (Fluke et al., 2021; Soneson et al., 2023), creating a detection bias in register data when interest lies in frequencies, prevalence, and trends. Survey data are often seen as the best way to evaluate these three aspects; however they may suffer from nonresponse biases—which lead to sample selection—recall, and other response biases, particularly when dealing with sensitive topics such as violence (Ellonen et al., 2024; Nelson et al., 2017). National studies can significantly contribute to addressing the limitations of survey data, as they involve large, representative samples that cover diverse populations offering more generalizable results. Furthermore, they employ standardized methodologies, that help minimize methodological variations and ensure consistency across different regions and populations (Fluke et al., 2021; Soneson et al., 2023).

However, only a few national studies have specifically addressed the association between pIPV and pCM. The most recent survey study by Miranda et al. (2021) utilized a large national sample (*N* = 19,684) of 12- to 16-year-old Chilean students and found that 58.7% and 39.4% of those adolescents who had been exposed to pIPV had also been physically maltreated during their lifetime and in the past year, respectively. Adolescents exposed to pIPV were four times more likely to experience pCM during their lifetimes and five times more likely during the past year compared to adolescents who were not exposed to pIPV. A previous survey conducted by Shen (2009) utilized self-reports from adolescents and found that the lifetime prevalence rate of co-occurring pIPV and pCM among Chinese college students (*N* = 1,924) was 37%.

Hamby et al. (2010) combined interview data from American children aged 10 to 17 years old with data from caregivers of children under 9 years old. The lifetime prevalence of co-occurring pIPV and pCM was 31.1%, with children exposed to pIPV being five times more likely to experience physical maltreatment during their lifetimes compared to non-exposed children. In the past year, the prevalence rate for co-occurrence was 17.6%, and children exposed to pIPV were four times more likely to experience maltreatment during that period compared to those without exposure. As the analysis did not examine the interviews of parents and children separately, and over half (54%, *N* = 4,549) of the interviews were obtained from caregivers, it is not possible to differentiate the influence of caregiver perspective on the prevalence of co-occurrence. Chan’s (2011) study suggests that prevalence rates are considerably lower when examined from the perspective of parents (lifetime prevalence 22.8% and past year’s prevalence rate 15.6%) highlighting the importance of using children’s self-reports.

Due to varying research designs and methodologies, comparisons between national studies are challenging. Furthermore, this study highlights the need to examine pCM within different conceptual frameworks due to variations in legal frameworks, child welfare systems, and health organizations across different countries. Corporal punishment is still legal in the United States and China, where many of the previous national studies have been conducted. In contrast, Finland and several other European countries have prohibited corporal punishment by law (Council of Europe, 2023), creating a unique context for understanding child maltreatment. This study uniquely positions us to investigate the co-occurrence of children’s self-reports on experiencing pCM and exposure to pIPV, providing valuable insights into their co-occurrence and informing policy and intervention efforts both within Finland and internationally.

## The Present Study

This study fills the previously described gaps in knowledge by examining the association between pIPV and pCM in a nationally representative sample of 12 to 13 and 15- to 16-year-old Finnish children. We use the χ^2^ test to examine to what extent children in homes where pIPV is present also experience pCM from their parents. Using the logistic regression model, we examine whether the connection between pIPV and pCM remains statistically significant after controlling for other known risk factors.

The specific research questions are as follows:

1. What is the prevalence rate of co-occurring pIPV and pCM in a nationally representative sample of Finnish school-aged children?
2. Is there a connection between pIPV and pCM after controlling for other known risk factors?

We use the term pCM when referring to physically violent acts toward children perpetrated by parents and pIPV when referring to physically violent acts between guardians.

## Methods

### Sample and Procedures

This study is based on data from the Finnish Child Victim Survey conducted in 2022 by Tampere University. The sampling was made by Statistic Finland as a two-phase stratified sampling based on regional state administrative agencies and the size of the schools. In Finland, most pupils attend these public schools, which are maintained by municipalities and have no selection procedure. Basic education is free and compulsory for children aged 7- to 18 years-old (Kortekangas et al., 2019).

First, the schools were contacted to request their participation and to name a contact teacher who received written instructions on how to conduct the survey. The contact teacher informed the children about the topic of the survey, their voluntary participation, and the option to stop answering at any point. This was also emphasized in the introduction of the survey, along with confidentiality and anonymity. The study was web-based, and the pupils completed it during a lesson in school in May 2022. The contact teacher was responsible for providing a secure, private, and calm space to answer the survey.

The children were the key persons in deciding whether to participate in the survey, and parents’ permission was not required according to Finnish National Board on Research Integrity (2023). It was also voluntary for the children to inform their parents after completing the survey. After completing the survey, the children were directed to a page that offered extra reading and tasks for those who finished early, so nobody could see how long it took for others to answer. The webpage included information on support organizations and chat rooms, which were aware of the themes and timeframe of the Child Victim Survey and ready to help if the children wanted to discuss the topics.

The original sample size was 23,049 sixth and ninth-grade pupils, and a total of 6,827 pupils completed the survey. Most of the attrition occurred at the school level, and once a school decided to participate, most of the students in the chosen classes answered the survey. This suggests that there is no reason to assume that children who have experienced violence have systematically failed to respond to the survey (Mathews et al., 2022). According to the dropout analysis, the declining schools were evenly distributed throughout Finland and therefore did not affect the generalization of the estimates. In addition, systematic attrition was assessed by comparing some key statistics of the data to other national surveys targeting the same age group in Finland and they were found to be similar. Missing data was analyzed and compared to the previous Child Victim Survey conducted in 2013 indicating no nonresponse bias.

Sixth-grade pupils accounted for slightly over half (54.6%, *n* = 3,654) of the respondents, and they were 12 to 13 years old. Ninth-grade pupils accounted for 45.5% of the respondents, and they were 14 to 16 years old (*n* = 3,034). Girls represented 51.4% (*n* = 3,408) of the sample, and boys represented 48.6% (*n* = 3,227).

### Measures

#### Physical Child Maltreatment

Our key outcome of interest was whether the children in the study had experienced pCM during the past 12 months. The Child Victim Survey covers a wide variety of questions related to violence against children in different environments, such as home and school. For the present study, we selected questions concerning pCM perpetrated by parents. The questionnaire did not specify the definition of parents in more detail. The measures are based on the Finnish translation of the Conflict Tactics Scale Parent-Child version by Straus et al. (1998). Physical maltreatment was measured with the following question: “Has your mother ever during arguing. . .” and the acts of violence were: “Pushed, shoved, or shook you angrily,” “Pulled your hair,” “Slapped you,” “Hit you with a fist,” “Hit you with something,” “Kicked you,” “Spanked you,” “Threatened you with a knife or gun,” and “Used a knife or gun.” The response options were “No,” and “Yes.” There were similar questions and response options concerning fathers. The outcome was 1 if the child answered yes to at least one form of pCM perpetrated by the mother or by the father, and 0 otherwise.

#### Physical Intimate Partner Violence

The children’s exposure to pIPV was measured based on a series of questions invented by a Norwegian social research institution (Mossige & Stefansen, 2007). The set of questions begins as follows: “Have you heard or seen that any of the following has happened to your mother in the last 12 months at your home?” The acts of physical violence were: “She has been pushed, shoved, or shook angrily,” “Her hair has been pulled,” “She has been slapped,” “She has been hit with a fist,” “She has been hit with something,” “She has been attacked with a knife,” “She has been threatened with a gun,” “She has been a target of some other kind of violence.” The response options were “Yes” and “No.” If any of the answers were “Yes,” there was a follow-up question about the perpetrator: “Who did these things to your mother?” and the response options were “Father,” “Stepfather or mother’s partner,” “Brother,” “Stepbrother,” “Sister,” “Stepsister,” and “Me.” For our analysis, we selected cases in which the mother had been the victim of physical violence perpetrated by the father, stepfather, or mother’s partner. The survey included similar questions and response options about violence against the father. When the father was the target, we selected cases in which the perpetrator was a mother, stepmother, or father’s partner. The child was seen to have been exposed to pIPV if she/he had heard or seen at least one form of physically violent act toward the mother or father.

#### Covariates

Previous research has identified several risk factors related to both pIPV and pCM. Many of these risk factors function by interfering with family dynamics and interaction. For example, children’s alcohol use and experiences with drugs often reflect other problems, such as behavioral issues, contributing to family stress and conflict, which can increase the likelihood of maltreatment (Chan, 2011; Guedes et al., 2016; Liel et al., 2022; Rada, 2014; Stith et al., 2009). We used these known risk factors as covariates. Child-related risk factors were sex assigned at birth (boy/girl), grade (sixth/ninth), nationality (Finnish/foreign), possible injury, disease, or special need (no/at least one injury, disease, or special need), substance abuse (no/has tried at least once), and experiences with alcohol (no/has tried at least once). Parent and family-related risk factors were the child’s history in foster care (no/yes), family structure (nuclear/other), parents’ foreign background (both parents born in Finland/at least one parent born somewhere else), parents’ education (primary/at least one parent with higher education), feeling comfortable discussing with parents (yes/no), parents knowing where and with whom the child spends time (yes/no), unemployment in family (both parents working/at least one parent unemployed), substance abuse in family (no/yes), frequent alcohol consumption in the family (no/yes), and emotional maltreatment by parents during past 12 months (no/yes).

### Statistical Analyses

We used descriptive analysis (frequencies and percentages) to describe the covariates and prevalence rates of pIPV and pCM (Table 1). The χ^2^ test and Fisher’s exact test were used to examine the prevalence rate of co-occurring pIPV and pCM and to examine which risk factors were statistically significantly connected to pCM. Logistic regression analysis was utilized to further examine the co-occurrence. We included only pIPV in the first model and added all the covariates that were, according to our χ^2^ test, statistically significantly associated with pCM to the second model in the logistic regression. Weighting coefficients were used to correct for attrition. The analyses were performed using SPSS version 25 by IBM.

**Table 1.** pIPV and Covariates by Physical Child Maltreatment and Total Sample.

Variables	Physical Child Maltreatment Past 12 months				Total	
	Yes		No			
	%	*n*	%	*n*	%	*n*
Total	7.2	307	92.8	3,951		
Child-related factors						
Sex assigned at birth						
Boy	5.3	95	94.7	1,695	48.6	3,227
Girl	8.6***	206	91.4	2,176	51.4	3,408
Grade						
Sixth	7.3	165	92.7	2,090	54.6	3,654
Ninth	7.1	139	92.9	1,817	45.4	3,034
Nationality						
Finnish	6.9	280	93.1	3,785	95.5	6,401
Foreign	17.3***	26	82.7	124	4.5	299
Injury, disease, or special need						
No	6.3	203	84.4	3,005	74.6	5,093
At least one	9.9***	104	78.2	946	25.4	1,732
Substance abuse						
No	6.8	280	93.2	3,840	3.0	200
Yes	23.6***	26	76.4	84	97.0	6,448
Experiences with alcohol						
No	5.9	174	94.1	2,795	69.5	4,547
Yes	10.6***	127	89.4	1,073	30.5	1,998
Foster care before						
No	7.1	298	92.9	3,888	99.1	6,615
Yes	16.7	4	83.3	20	0.9	60
Parent and family-related factors						
Family structure						
Nuclear	6.2	187	93.8	2,852	69.8	4,713
Other	9.7***	116	90.3	1,078	30.2	2,041
Parents’ foreign background						
Both parents born in Finland	6.6	246	93.4	3,485	84.6	5,774
At least other parent born somewhere else	11.6***	61	88.4	465	15.4	777
Parents’ educational level						
Primary	7.5	12	92.5	147	6.7	278
Higher	6.7	575	93.3	2,354	93.3	3,839
Feeling comfortable discussing with parents						
Yes	6.0	238	94.0	3,761	93.8	6,289
No	28.4***	66	71.6	166	6.2	414
Parents know where and with whom the child spends time with						
Yes	6.9	284	93.1	3,823	98.4	6,429
No	28.3***	13	71.7	33	1.6	104
Unemployment in the family						
Both parents working	7.1	296	92.9	3,876	98.0	6,685
At least one parent unemployed	12.9*	11	87.1	74	2.0	140
Substance use in the family						
No	7.0	293	93.0	3,885	99.1	6,554
Yes	32.0***	8	68.0	17	0.9	59
Alcohol consumption often in the family						
No	7.1	132	92.9	1,723	78.2	2,792
Yes	12.7***	62	87.3	427	21.8	777
Family violence						
Physical Intimate partner violence during the past 12 months						
No	6.7	281	93.3	3,922	98.9	6,747
Yes	47.3***	26	52.7	29	1.1	78
Emotional maltreatment by parents during the past 12 months						
No	0.9	25	99.1	2,812	60.9	2,832
Yes	20.4***	267	79.6	1,042	39.1	1,818

*Note.* Fisher’s exact test was used for variables foster care before, parents know where and with whom the child spends time, and substance use in the family due to small cell frequencies. The χ2 test was used for the other variables. *p*IPV = physical intimate partner violence.

* *p* < .05. ***p < .001.

## Results

A total of 307 children reported experiencing pCM during the past 12 months, and the prevalence of pCM was 7.2%. In total, 78 children had been exposed to pIPV in the past 12 months, and the prevalence rate of pIPV was 1.1%. All covariates except the child’s age, the child’s previous foster care and parents’ education were statistically significantly (*p* < .001) associated with pCM. In total, 47.3% of those who reported being exposed to pIPV during the past 12 months also reported being physically maltreated by their parents during the past 12 months.

According to the logistic regression analysis, pIPV was still associated with pCM when other known risk factors were controlled for (Table 2). Children exposed to pIPV were more likely to be physically maltreated (OR = 2.57, *p* = .02) by their parents during the past 12 months than children who were not exposed to pIPV. The decrease in the *p*-value (from <.001 to .02) indicates that the covariates included in the second model explain some of the variances in the relationship between pIPV and pCM. Living in a family where parents engage in emotional maltreatment or consume alcohol often as well as feeling uncomfortable discussing with parents, remained statistically significantly associated with a higher likelihood of experiencing pCM in this sample. However, pIPV still emerged as a significant predictor.

**Table 2.** Relative Odds Ratios and 95% Confidence Intervals Based on Logistic Regression of the Association Between pIPV and pCM (Step 1) Controlling for Known Risk Factors (Step 2).

	Step 1		Step 2	
Risk Factors	OR	[95% CI]	OR	[95% CI]
Intimate partner violence				
No	1.0		1.0	
Yes	7.00***	[3.46, 14.17]	2.57*	[1.16, 5.68]
Child-related factors				
Gender				
Boy			1.0	
Girl			1.02	[0.68, 1.52]
Nationality				
Finnish			1.0	
Foreign			2.13	[0.79, 5.78]
Injury, disease, or special need				
No			1.0	
At least one			0.16	[0.79, 1.71]
Substance abuse				
No			1.0	
Yes			0.53	[0.26, 1.07]
Experiences with alcohol				
No			1.0	
Yes			1.10	[0.76, 1.59]
Parent and family-related factors				
Family structure				
Nuclear			1.0	
Other			1.39	[0.96, 2.00]
Parents’ foreign background				
Both parents born in Finland			1.0	
At least other parent born somewhere else			1.05	[0.59, 1.89]
Feeling comfortable discussing with parents				
Yes			1.0	
No			3.11***	[1.88, 5.14]
Parents know where and with whom the child spends time with				
Yes			1.0	
No			1.45	[0.43, 4.92]
Unemployment in the family				
Both parents working			1.0	
At least one parent unemployed			1.07	[0.40, 2.87]
Substance use in the family				
No			1.0	
Yes			3.13	[0.89, 11.10]
Alcohol consumption often in the family				
No			1.0	
Yes			1.49*	[1.01, 2.21]
Emotional maltreatment by parents during the past 12 months				
No			1.0	
Yes			27.67***	[13.83, 55.36]
Sample size	2,072		2,072	

*Note.* The dependent variable, pCM was coded 0 = no self-reported experiences of pCM during past 12 months and 1 = has reported at least one experience of pCM during past 12 months. pCM = physical child maltreatment; pIPV = physical intimate partner violence.

* *p* < .05. ***p < .001.

As emotional maltreatment is so often present in pCM, we performed a sensitivity analysis to ensure that the model accurately explains physical maltreatment without emotional maltreatment mixing the results. The analysis was conducted similarly, but emotional maltreatment was removed from the model, and as a result, OR increased to 3.72 (*p* < .001) and more of the risk factors remained statistically significant. However, the main result remained the same, and pIPV was significantly associated with pCM.

## Discussion

The present study adds further support to the knowledge that there is a robust connection between pIPV and pCM (Boeckel et al., 2014; Casanueva et al., 2009; Seon et al., 2022; Sijtsema et al., 2020; Skafida et al., 2022). In this study, almost half of the children who had been exposed to pIPV also experienced pCM during the past 12 months; children exposed to pIPV were almost three times more likely to experience pCM compared to children who were not exposed to pIPV. Furthermore, this study adds support to the poly-victimization model (Finkelhor et al., 2007), highlighting the need for a comprehensive approach to the complex experiences of violence in childhood families. It also provides insight into the ongoing debate on the intergenerational transmission of violence, highlighting that intergenerational continuity of family violence may include more than just the transmission of one type of violence (i.e., child maltreatment from one generation to another). Violence that occurs across two generations, whether it remains the same type or changes form, can be understood to be intergenerational as it impacts at least two generations (Buchanan et al., 2023; Cochran et al., 2017; Haselschwerdt et al., 2019; Hou et al., 2016).

This study further contributes to the existing literature by providing new insights into co-occurring physical violence in families. Previous research has focused on examining the association between IPV and pCM combining physical and emotional IPV (Ahmadabadi et al., 2018; Seon et al., 2022), making it impossible to differentiate the influence of emotional IPV and pIPV on the co-occurrence. In this study the association between pIPV and pCM remained statistically significant even after controlling for other risk factors, indicating that pIPV itself contributes independently to the likelihood of pCM.

Furthermore, according to the sensitivity analysis, the association between pIPV and pCM is significant, regardless of emotional IPV. Although physical family violence is often accompanied by emotional family violence, emotional family violence can occur independently (Finkelhor et al., 2007; Hamby et al., 2010). Concentrating on physical violence in the family allows for a more nuanced analysis and interpretation of research findings, facilitating evidence-based policymaking, program development, and evaluation. Tailored prevention and intervention strategies are essential for addressing emotional and physical maltreatment effectively. Understanding the risk factors, dynamics, and consequences specifically associated with physical maltreatment enables targeted prevention efforts and interventions aimed at reducing the occurrence of physical family violence and mitigating the impact of abuse on children and families (Gilbert et al., 2009; Harper et al., 2018; MacDonell, 2012; Yohros, 2023).

One of the main strengths of the present study is the relatively large, nationally representative sample. Therefore, it provides new insight into the connection between pIPV and pCM in the general population (Ellonen et al., 2024; Tiyyagura et al., 2018). The findings indicate that pCM is more common than pIPV in the studied population. Although the prevalence of witnessed pIPV seems low, it is consistent with the previous Child Victim Survey (Fagerlund et al., 2013). A significant finding is that the co-occurrence of pIPV and pCM is high, even at the national level. This result is worrying since it means that pIPV and pCM co-occur significantly even in families that are not in the high-risk group but represent the “average” families in society. The high co-occurrence rate underscores the need for integrated policies and interventions targeting both forms of physical violence, family dynamics, and parental support, as well as parents’ attitudes toward corporal punishment to effectively protect children and support families (Copp et al., 2019; Ellonen et al., 2015; Harper et al., 2018; Howell et al., 2016).

Moreover, pIPV and pCM often occur behind closed doors, and there might be no signs of violence in the child or in the family. This can be the case even though pCM that co-occurs with pIPV tends to be more severe than maltreatment in the absence of pIPV (Sijtsema et al., 2020; Skafida et al., 2022). Hence, recognizing physical family violence can be challenging (Laajasalo et al., 2023). Additionally, pCM has become more prevalent both nationally (Mielityinen et al., 2023) and internationally (Lee & Kim, 2023). These findings emphasize the need to further examine physical family violence as research results contribute to the planning and implementation of both preventive and intervention services. It is essential to prevent both pIPV and pCM from happening, not only from an economic point of view but also in terms of preventing human suffering (Harper et al., 2018; Howell et al., 2016).

This study provides a unique context for examining pCM, as corporal punishment is legally prohibited in Finland (Council of Europe, 2023). Prohibiting corporal punishment has also led to a change in attitudes toward physical maltreatment and raised caregivers’ awareness of the harmful effects of abuse on children’s well-being. These changes potentially influence prevalence rates and perceptions compared to studies conducted in countries where corporal punishment is accepted. Furthermore, Finland is culturally quite different from the countries cited in this study, and variations in culture might also explain some of the variations in the co-occurrence. Therefore, it is essential to further examine the co-occurrence of pIPV and pCM in different countries and contexts. Additionally, attitudes toward corporal punishment and family violence, as well as societal norms regarding acceptable behavior within family dynamics, significantly influence prevalence rates of pCM (Copp et al., 2019; Ellonen et al., 2015). Understanding these attitudes is crucial for developing effective interventions and policies aimed at reducing pCM and promoting child well-being.

This study is among the few to examine the connection between pIPV and pCM in the general population from the children’s perspective. Furthermore, this study did not require consent from the parents of the children to participate. In research settings that require parental consent for children to participate, children who have experienced parental maltreatment may be excluded from the study, thereby preventing them from reporting their experiences. The explanatory power and strength of the findings of this study are enhanced by the fact that the most accurate information about maltreatment and experiences of violence comes directly from the children themselves (Fluke et al., 2021; Laajasalo et al., 2023; Mathews et al., 2022; Soneson et al., 2023). Furthermore, since the reference period covered only the past year, recall bias in this study is considered to be minimal, unlike in studies examining child maltreatment by asking adults about their childhood experiences. These reports may be influenced by memory and mood (Nelson et al., 2017).

## Limitations and Future Research

The results of this study should be considered in light of its limitations. First, using cross-sectional data, we are prevented from presenting any causal conclusions from the connection between pIPV and pCM. Longitudinal research is needed to examine causal relations. Second, as the prevalence of both pIPV and pCM was relatively small, though robust and comparable to other national studies and previous findings, we were unable to conduct separate analyses for mothers and fathers. With larger samples, it is possible to provide valuable information about parents’ different roles in perpetrating both pIPV and pCM and to offer a more complete evaluation of the connection. Third, some information might have been lost by dichotomizing the risk factors and experiences of pIPV and pCM in the analysis. However, this is a commonly used procedure in the social sciences. Finally, retrospective self-reports of pCM might be affected by desirability bias and inaccurate recall of childhood experiences, though the reference period in this study was relatively short. Because this study measures the subjective perceptions of pCM, the acts cannot be verified.

## Implications for Practice and Conclusions

With this evidence, public policy and practitioners should address pIPV and pCM hand-in-hand. However, despite all the research revealing the connection, to some extent, social and health services fail to appreciate that the presence of pIPV should be an indicator of the importance of assessing the needs of children for both support and protection when living in the same household as the victim (Ahmadabadi et al., 2018; Casanueva et al., 2009; Miranda et al., 2021).

The results of this study suggest that preventing pIPV itself may be the most direct way to reduce pCM. As pCM generally happens behind closed doors (Skafida et al., 2022), any sign or indicator of the existence of pIPV in families should be considered a matter of concern. For example, in Finland, the National Institute for Health and Welfare recommends the use of the IPV filter and mapping form in all social and health services as part of mapping the basic situation of each family (October & Laitinen, 2022). If a person reports any pIPV in the family where there are minors present, the professional is obliged to make a child welfare report (Child Welfare Act, 2010/88). However, the implementation of this form is only a recommendation and it is not widely used in basic health care or is utilized only in early childhood, ruling out older children and their exposure to pIPV (October & Laitinen, 2022). As all children under school age are entitled to maternity and child health clinic services and school-aged children are entitled to school health care in Finland (Kortekangas et al., 2019), professionals in these organizations could be the key persons to detect the signs of family violence.

In addition to preventive actions, integrated intervention efforts should address multiple forms of victimization simultaneously (Finkelhor et al., 2007; Jouriles et al., 2008; October & Laitinen, 2022), and family programs should take pIPV more into account. To date, little is known about the treatment and prevention of pCM in the context of pIPV (Jouriles et al., 2008), especially in Finland. Furthermore, when planning for prevention and intervention, child-, parent-, and family-related risk factors should be considered closely, as they are strong predictors of pCM alongside pIPV (Gilbert et al., 2009; Liel et al., 2022; Sijtsema et al., 2020).

In conclusion, an integrated and holistic approach to assessing co-occurring pIPV and pCM improves the understanding of the connection between different victimizations in the family. Utilizing children’s own perspectives is vital, as the most accurate information about maltreatment comes directly from the children themselves. Early identification and intervention of both pIPV and pCM are needed to interrupt the sequence to effectively protect children and support families. From the perspective of a child, all forms of family violence should be taken seriously, as childhood is a highly vulnerable developmental stage for all kinds of adverse experiences.

## Supplemental Material

sj-docx-1-jiv-10.1177_08862605241289476 – Supplemental material for Is Physical Intimate Partner Violence a Risk Factor for Physical Child Maltreatment in a Nationally Representative Sample of Finnish School Children? Supplemental material, sj-docx-1-jiv-10.1177_08862605241289476 for Is Physical Intimate Partner Violence a Risk Factor for Physical Child Maltreatment in a Nationally Representative Sample of Finnish School Children? by Laura Annika Mielityinen, Noora Ellonen and Monica Fagerlund in Journal of Interpersonal Violence
