# Supplementary material for: Is Physical Intimate Partner Violence a Risk Factor for Physical Child Maltreatment in a Nationally Representative Sample of Finnish School Children?
Source: J Interpers Violence. 2024 Oct 23;40(17-18):4325–45. doi: 10.1177/08862605241289476 (PMC12308040; doi:10.1177/08862605241289476)
Supplement: sj-docx-1-jiv-10.1177_08862605241289476 – Supplemental material for Is Physical Intimate Partner Violence a Risk Factor for Physical Child Maltreatment in a Nationally Representative Sample of Finnish School Children? [file sj-docx-1-jiv-10.1177_08862605241289476.docx]

**Supplementary file 1.**

This supplementary file contains detailed diagnostics to support the findings of the logistic regression analysis.

**Multicollinearity Checks**

**Correlation coefficients, VIF (Variance Inflation Factor)** **and tolerance**

Multicollinearity is a concern when there are strong correlations (close to +1 or -1) between the dependent variables. We Computed a correlation matrix and according to it the correlations varied between +0,25 and -0,27 besides the variables concerning child’s and parents’ nationality where correlations coefficient was 0,44. As the correlation is still mild, these variables were included.

VIF indicates how much the variance of the regression coefficient is inflated due to multicollinearity. A VIF value greater than 10 indicates high multicollinearity (some use a threshold of 5).

Tolerance is the inverse of VIF and indicates the proportion of variance in the predictor that is not explained by other predictors. A tolerance value close to 1 indicates little multicollinearity and a tolerance value close to 0 indicates high multicollinearity.

According to our examination the VIF values varied between 1,02-1,15 and the tolerance between 0,87-0,99.

As all VIF values are below the threshold of 10 (and even below 5) and the tolerance values are all close to 1, multicollinearity is not a concern for the predictors in this analysis.

**Model Fit Statistics**

**Overall Model Fit**

The metrics used to assess overall model fit were -2 Log Likelihood, Akaike Information Criterion (AIC), Bayesian Information Criterion (BIC)).

Metrics for model 1

- -2 Log Likelihood: 1130,850
- AIC: -2257.700
- BIC: -2246.430

Metrics for model 2

- -2 Log Likelihood: 846,354
- AIC: -1662.708
- BIC: -1578.183

In our logistic regression analysis, the log likelihood values indicate a reasonably good fit of the model to the observed data, though the value is lower in model 2. However, the AIC and BIC values suggest that the model provides a good balance between fit and complexity. Lower AIC and BIC values indicate better model fit and parsimony, supporting the adequacy of the logistic regression model in explaining the relationship between the predictor variables and the outcome.

**Pseudo R-squared**

To further evaluate the model fit, we examined the Pseudo R-squared values. Pseudo R-squared is a statistical measure that quantifies the proportion of variation in the dependent variable that is explained by the independent variables in a logistic regression model. A Pseudo R-squared value close to 0 indicates that the model does not explain much of the variation in the dependent variable. A pseudo R-squared value close to 1 suggests that the model explains a large proportion of the variation in the dependent variable.

The Nagelkerke's R-squared values for our logistic regression models were 0,026 for the first model and 0,323 for the second model. The Cox & Snell R-squared values for our logistic regression models were 0,011 for the first model and 0,138 for the second model. The increase in both R-squared values from the first to the second model indicate a substantial improvement in model fit, with the second model explaining a larger proportion of the variance in the outcome variable. Despite relatively low R-squared values, these findings highlight the importance of the additional predictor variables or model adjustments in enhancing the explanatory power of the logistic regression model.

**Hosmer-Lemeshow Test**

We used the Hosmer-Lemeshow test to assess the goodness of fit of our logistic regression model.

For Model 1, the Hosmer-Lemeshow chi-square statistic was 0 with 0 degrees of freedom, indicating no p-value was calculated.

The test yielded a Hosmer-Lemeshow chi-square statistic of X = 8,834, with 8 degrees of freedom for the second model. The associated p-value was p = 0,356

Given that p > 0.05 for Model 2, in this case, the logistic regression model is considered to have a good fit, indicating that the predicted probabilities closely match the actual outcomes across different groups of predicted probabilities.
